# Effective Delivery of siRNA-Loaded Nanoparticles for Overcoming Oxaliplatin Resistance in Colorectal Cancer

**DOI:** 10.3389/fonc.2022.827891

**Published:** 2022-02-21

**Authors:** Yue Zhou, Qing Zhang, Minjia Wang, Chengzhi Huang, Xueqing Yao

**Affiliations:** ^1^ The Second School of Clinical Medicine, Southern Medical University, Guangzhou, China; ^2^ Department of Gastrointestinal Surgery, Ganzhou Municipal Hospital, Ganzhou, China; ^3^ Department of Gastrointestinal Surgery, Department of General Surgery, Guangdong Provincial People’s Hospital, Guangdong Academy of Medical Sciences, Guangzhou, China; ^4^ Department of Gastrointestinal and Anorectal Surgery, The First People’s Hospital of Zhaoqing, Zhaoqing, China; ^5^ School of Medicine, South China University of Technology, Guangzhou, China

**Keywords:** oxaliplatin, chemoresistance, siRNA delivery, colorectal cancer, ATP7A

## Abstract

Chemotherapy resistance represents a formidable obstacle in advanced or metastatic colorectal cancer (CRC) patients. It is reported that ATPase copper transporting alpha (ATP7A) plays an important role in chemotherapy resistance in CRC. Here, we identified ATP7A as a potentially key gene of OXA resistance in CRC. The patients with higher expression of ATP7A tended to have platinum drug resistance. While the lower expression of ATP7A by siRNA knockdown resulted in enhancement of OXA sensitivity and increased OXA-induced apoptosis. Further, we demonstrated a novel and safe strategy to increase CRC chemosensitivity by delivering siRNA into tumor cells *via* a novel nanoparticle, DAN. In summary, our study provided a novel nanocarrier-based delivery of ATP7A to interfere in a key gene of chemo-resistance in CRC, which may be a novel therapeutic strategy to overcome chemotherapy resistance in CRC.

## Introduction

Although the rapid development of cancer therapeutic methods, colorectal cancer (CRC) is still the third most common cancer in the world (1). The early-stage CRC patients may benefit from radical surgical resection with a higher disease-free survival (DFS) rate of nearly 90% (2, 3). However, the advanced or metastatic CRC patients might not have the chance to accept the radical surgical resection (3, 4). As a result, chemotherapy, targeted therapy, or immunotherapy might be a better preferable therapeutic method for those patients.

Oxaliplatin (OXA), a Pt-based chemotherapy drug, combined with 5-fluorouracil (5-Fu) or capecitabine was introduced for first-line chemotherapy methods for advanced CRC patients (5, 6). The FOLFOX protocol (OXA combined with 5-Fu) or CapeOx protocol (OXA combined with capecitabine) were significantly improved the clinical prognoisis (7–9). However, the overall survival (OS) or DFS for advanced CRC patients remained low. One of the most important reasons for poor prognosis might be the resistance against Pt-based chemotherapy (10). New studies are raising more and more questions nowadays about the mechanism based on chemotherapy resistance. Researches found that the primary mechanisms of platinum resistance included drug accumulation, the ability of DNA or RNA repair efficiency, and self-detoxification of cells (11). Of those, reduction in drug accumulation is one of the most generally acknowledged mechanisms of acquired OXA resistance (12).

ATPase copper efflux transporter A (ATP7A) is a member of intracellular copper transporters, ubiquitously expressed and transports copper out of the cell to maintain cellular copper homeostasis (13, 14). Additionally, ATP7A is also found to be one of the key regulators of intracellular platinum levels by delivering platinum to the extracellular space, reducing intracellular drug accumulation, thus compromising therapeutic efficacy (15–17). Furthermore, it has been reported that ATP7A is related to the resistance of platinum-based chemotherapy in various kinds of cancers, such as CRC and breast cancer (14, 18). Therefore, the development of the strategy to specifically knockdown ATP7A is considered to be a feasible treatment for colorectal cancer.

Gene interference, a relatively novel technique for switching off individual genes by delivering small interfering RNA (siRNA), has become an anti-tumor therapy with broad application prospects (19). The siRNA aimed to knockdown a targeted gene by delivering a specific sequence into a cell (20). Owing to the instability, poor blood circulation, and low uptake efficiency of free siRNA, the key to gene interference is how to transport therapeutic genes and drugs safely and efficiently to the tumor site (21, 22). Therefore, it is significant to find an effective delivery to transfer siRNA into cancer cells for anti-tumor treatment (23). (1,2-Dioleoyl-3-trimethylammonium-propane) DOTAP-assisted nanoparticles (DANs) could overcome the challenge of siRNA delivery, which helps effectively deliver siRNA, mRNA, or plasmid into cancer cells (24–26).

In the current research, we encapsulated siRNA using DANs and confirmed that DAN-siATP7A could be effectively downregulated the target gene *in vitro* and *in vivo*. As expected, after systemic injection of DAN-siATP7A into the model mice of CRC, the antitumor efficacy of OXA has been significantly enhanced with the size and weight of the tumor decreasing dramatically. Meanwhile, in terms of the weight curve, blood index, and organ index of the mice, combination therapy is relatively safe with few or minimal undesired systematic toxic effects. These results verified our hypothesis that nanocarrier-mediated interference of ATP7A could overcome oxaliplatin resistance in CRC. The combination use of nanoparticles and OXA represents a potential alternative therapy method against advanced cancer.

## Materials and Methods

### Patients and Tissues Specimens

The tumor tissue specimens of CRC patients obtained from palliative or radical surgical resection were randomly chosen between 2015 to 2016 from Guangdong Provincial People’s Hospital (Guangdong Academy of Medical Sciences). The inclusion criteria were: 1) All the patients were underwent palliative or radical surgical resection, and was pathologically diagnosed as adenocarcinoma. 2) All the included patients were received standardized OXA-based chemotherapy protocol. The exclusion criteria were: 1) pathologically diagnosed as gastrointestinal stromal tumors or squamous carcinoma.

Tumor stages were defined according to the 8th edition of the TNM staging system. To evaluate the therapeutic effect of OXA-based chemotherapy, we analyzed the results of radiology or pathology examination and referred to the following criteria: partial response (PR), complete response (CR), stable disease (SD), and progressive disease (PD). In our study, the OXA-response was defined as PR or CR, and the SD, PD were considered as OXA-resistance. All specimens were obtained with written informed consent and the study was approved by the Institutional Review Board of Guangdong Provincial People’s Hospital under the grant number: GDREC2019504H(R2).

### Immunohistochemistry and Hematoxylin-Eosin Staining

To further evaluate the potential association of ATP7A with OXA-resistance in CRC, immunohistochemistry (IHC) analysis was performed in our research as previously described (27). In brief, the primary antibody against ATP7A was purchased from BBI LIFE Science Corporation, (D161470, BBI, China). Two qualified pathologists independently analyzed the IHC results of the ATP7A protein expression based on the percentage of positive cells and staining intensity of the cytoplasm and nucleus. The score of percentage in positive cells was defined as 0 (<10%), 1 (10–25%), 2 (25-50%), and 3 (>50%), and the score of staining intensity were defined as 0 (negative staining), 1 (weakly staining), 2 (moderately staining), and 3 (strongly staining). The sum of the two scores was regarded as the final IHC score. High expression of ATP7A was defined as an IHC score from 4 to 6, while 0 to 3was regarded as low expression. We also performed Hematoxylin-Eosin (HE) staining in tumor tissues and viscus tissues from nude mice following the instruction of the manufactory (Biosharp, China).

### Cell Culture and Transfection

We obtained human CRC cell lines, HCT116 and LOVO, from the iCell Bioscience (iCell, Shanghai, China). These cells were cultured in RPMI-1640 medium (BL303A, Biosharp, China) containing 10% fetal bovine serum (FBS, BL201A, Biosharp, China) supplemented with 1X penicillin-streptomycin solution (BL505A, Biosharp, China) at 37°C in a humidified incubator containing 5% CO2.

The siATP7A and cy5-labeled siNC were synthesized in Suzhou Gene Pharma and transfected into HCT116 and LOVO cells at a final concentration of 50 nM to knockdown ATP7A messenger RNA. The Lipofectamine 3000 (Invitrogen, USA) was utilized in the transfection procedure. Meanwhile, western- blotting and qPCR were utilized to evaluate the transfection efficacy following the manufacturer’s protocol. The target of siATP7A sequence is as follows: 5’- UAUCCUAUGGUUAAACCUCUG-3’, The scrambled (NC) siRNA sequence is 5’-UUCUCCGAACGUGUCACGU-3’.

### Bioinformatic Analysis

The expression and Kaplan-Meier survival analysis of ATP7A in CRC patient cohorts were performed in Gene Expression Profiling Interactive Analysis (GEPIA, http://gepia.cancer-pku.cn/index.html) database (28).

### Western-Blot

The Western Blot was performed as previously described (27). In brief, total protein of cells was extracted with RIPA buffer (Beyotime, China) supplemented with protease inhibitor, phenylmethylsulfonyl fluoride (PMSF, Beyotime, China) at 4°C, 14000 rpm for 20 min. BCA assay (Fudebio, China) was used for protein quantification. The protein was isolated by electrophoresis using 10% SDS-polyacrylamide gel (PAGE) and then transferred to the PVDF membrane (Immobilon P, Millipore, USA). 5% fat-free milk (Beyotime, China) in Tris-buffered saline with Tween-20 (TBST) was used to block nonspecific binding sites at room temperature for 1h. Then, the membranes were immunoblotted with primary antibody overnight at 4°C: anti-ATP7A (1:1000, D161470, Sangon Biotech, China) and anti-GAPDH antibody (1:1000, AP0066, Bioworld Technology, USA). After being washed with TBST 3 times, blots were incubated with secondary peroxidase-conjugated antibodies, the goat anti-rabbit secondary antibody (1:5000, BS13278, Bioworld Technology, USA) for 1 h at room temperature. The membranes were performed with an enhanced chemiluminescence system and scanned by the Syngene Imaging System (Frederick, USA).s

### Quantitative Real-Time Polymerase Chain Reaction

Total RNA of CRC cells was extracted with TRIzol RNA Isolation Reagents (ThermoFisher Scientific, USA) as previously described. The 500ng of extracted RNA was reverse-transcribed into complementary DNA (cDNA) using the First-strand cDNA Synthesis Kit (K1072, Apexbio, USA) following the manufacturer’s protocol. qPCR was performed on the LightCycler 480 (Roche, Swiss) instrument by using 2X SYBR Green PCR Mix (K1070, Apexbio, USA). ΔΔCT was calculated to analyze the results of qPCR and data from each experiment were normalized to the expression of a control gene (GAPDH). The following primers were used:

ATP7A, forward 5′-TGACCCTAAACTACAGACTCCAA-3′ and reverse: 5′-CGCCGTAACAGTCAGAAACAA-3′; GAPDH, forward 5′-TCAACGGATTTGGTCGTATTGGGCG-3′ and reverse 5′-CTCGCTCCTGGAAGATGGTGATGGG-3′.

### Cell Viability Assay

For detection of OXA cytotoxicity (A8648, Apexbio), the CRC cells were seeded at a density of 3,000 per well into 96-well plates and cultured in a complete medium containing different concentrations of OXA for 48 h. Then, the Cell Counting Kit-8 (CCK-8) detection Kit (K1018, Apexbio, USA) was used to assess the inhibitory concentration of OXA. After continuing incubation for 4 h, the absorbance in 450 nm was detected *via* a microplate reader (BIO-Tek SYNERGY H1, USA). For detection of cell viability, 1,000 cells were seeded into each well of the 96-well plate with OXA of the 50% inhibitory concentration (IC50). CCK-8 solution was added to the wells at the time point of 0, 24, 48, and 72 h. The absorbance of stained cells was measured at 450 nm after continuing incubation for 4 h.

### Apoptosis Detection

Cell Apoptosis Detection Kit (E-CK-A217, Elabscience, China) was utilized in apoptosis detection assays. The cultured cells were digested by trypsin, resuspended in 500 μL of binding buffer, and stained with Annexin V-FITC and propidium iodide (PI) for 15 min in dark. All samples were detected by a flow cytometer (BD FACS Verse, BD bioscience, USA) and the results were analyzed using the FlowJo 10.6 software (BD bioscience, USA).

### Wound-Healing Assay

Cells in each group were seeded into a 6-well plate at a density of 2 ×10^6^ per well to confluence After being cultured in pure RPMI-1640 medium without FBS, two straight wounds were made by a 200ul pipette tip (ThermoFisher Scientific, USA) in each well. At the time point of 0h, 24h, and 48 h, the migration status was photographed at the same location of wells using a light microscope (Olympus BX63, Japan) The result was analyzed by the ImageJ software.

### Colony Formation Assay

Cells in each group were seeded into 6-well plates at a density of 1000 per well and continuously cultured for 2 weeks. The RPMI-1640 complete culture medium was replaced every 3 days. At the end of the experiment, 0.1% crystal violet (PH1322, Phygene Scientific, China) was used to stain the colonies. The number of colonies was counted by ImageJ software (29, 30).

### Migration Assay

For migration assay, 8 μm-pore transwell chambers (Corning Costar, USA) were used without Matrigel. Each well of 24-well plates was filled with the RPMI-1640 complete culture medium containing 10% FBS. Then, approximately 1 × 10^5^ cells in 500ul FBS-free medium were pipette into the upper chamber slowly. The incubation time is 48 h for HCT116 and LOVO. After being washed by PBS 3 times, cells on the upper surface of the chamber were removed lightly using a cotton swab. Then, the cells migrated to the lower surface of the chamber were fixed using 4% paraformaldehyde (PFA). At the end of the experiment, cells were stained with 0.1% crystal violet solution and counted and analyzed by ImageJ software.

### Construction of Nanoparticles

Nanoparticles (DANs) encapsulating siRNA were prepared through a double emulsion solvent evaporation technique as previously described (24, 31). The PEG5k-b-PLGA11k (molar ratio: LA/GA=75/25) was purchased from Kelanbio (Guangzhou, China) and 1,2-Dioleoyl-3-trimethylammonium-propane (DOTAP) was purchased from Topscience (Shanghai, China) (24). Firstly, 25 μL DNase/RNase-free water containing 5 OD siRNA and 500 μL chloroform containing DOTAP (1 mg) and PEG5K-b-PLGA11K (25 mg) were emulsified for 1 min at 80W in an ice bath by sonication using Sonics Materials™ ultrasonic Processor VCX130-220V (Sonics & Materials, Inc, Newtown, USA). Then, 5 mL DNase/RNase-free water was added to the primary emulsion and further emulsified for 1 min. Finally, the organic solvent was removed with a rotary evaporator RV10 auto control (IKA, Germany). The diameter of DANsiATP7A was monitored at 25°C with Malvern Zetasizer Nano ZSE (Worcestershire, UK).

### Cellular Uptake of DANsiRNA *In Vitro*

Cy5-siNC was encapsulated into DAN (DANCy5-siNC) to investigate cellular uptake of DAN by confocal laser scanning microscopy (CLSM). Cells were seeded into an observation dish for 24h and then incubated with DANCy5-siNC or free Cy5-siNC for 8 h. After the cell nucleus stained with DAPI (Beyotime, China), samples were photographed by Carl Zeiss LSM880 confocal microscope.

### Establishment Mice CRC Model

The nude mice (6-8 weeks old) were randomly allocated to control and experimental groups, and the mice were all bred in specific pathogen-free (SPF) conditions. Mice were injected subcutaneously with 5×10^6^ HCT116 cells. The tumor volume was calculated using the formula: Tumor Volume (mm^3^) = (longest diameter) * (shortest diameter)^2 * 0.5. When the size of the tumor reaches 150mm^3^, and the mice were randomly divided into 4 groups (PBS, free OXA, DAN-siATP7A, and combination use of DAN-siATP7A and OXA). After treatments, the tumors, organs, and blood from mice were dissected for further analysis. The animal experiment was proved by the Institutional Review Board of Guangdong Provincial People’s Hospital under the grant number: GDREC2019506A.

### TUNEL Staining for Apoptosis

Terminal deoxynucleotidyl transferase-mediated nick end labeling (TUNEL) assay is applied to determine the apoptotic cells. The CRC samples of mice were fixed in 4% PFA for 24 h, subsequently embedded in paraffin, and sliced (4–5 μm). The TUNEL staining was performed using the DAB (SA-HRP) TUNEL Cell Apoptosis Detection Kit (Servicebio, Wuhan, China) following the manufacturer’s protocol. Streptavidin labeled with horse-Radish peroxidase (HRP) (SA-HRP) was then detected the biotin-labeled DNA terminal. Finally, HRP substrate mixture (DAB) was added for color reaction, so that the apoptotic cell nucleus was dyed brown and yellow, which can be detected by the ordinary light microscope. The rate of apoptosis was displayed as the ratio of TUNEL positive nuclei to hematoxylin-stained nuclei.

### *In-Vivo* Toxicity Analysis

To further evaluate the *in vivo* toxicity of DAN nanoparticles, the crucial organs of the mice, such as the heart, liver, spleen, lung, kidney, and brain, were dissected for HE staining. Two experienced pathologists were invited to analyze the potential toxicity of the nanoparticles through the slides. Blood samples of mice have been collected to analyze the concentration of alanine aminotransferase (ALT), aspartate aminotransferase (AST), glutamyl transferase (GGT), blood urea nitrogen (BUN), Creatinine (Cr), and uric acid (UC) according to the manufacture’s instructions (Nanjing Jiancheng, China).

### Statistical Analysis

Statistical analysis was performed using Prism 9.0 (GraphPad, La Jolla, CA, USA) and Stata 15.0 (Stata Corp, USA). Data are presented as mean ± standard deviation (SD). The difference between the two groups was analyzed by the Student’s *t-*test or unpaired *t-*test. Comparison of three or more groups were using the one-way ANOVA analysis. The experiments had been performed in triplicate. A p<0.05 was considered as statistical significance.

## Results

### Implications of ATP7A Expression and Clinical Characteristic

**Figure 1:**
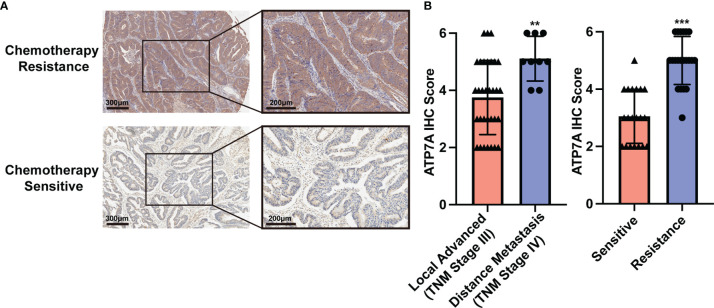
Implications of ATP7A expression and clinical characteristics. **(A)** Expression of ATP7A in OXA resistance and OXA responder of CRC patients. **(B)** IHC staining scores of chemotherapy resistance patients and chemotherapy responders. **P < 0.01 and ***P < 0.001 when compared to the control group.

In our cohort, a total of 41 advanced CRC cases were included and received standard eight-cycle OXA-based chemotherapy (FOLFOX or CapeOx protocol). 20 CRC patients benefit from the chemotherapy, while 21 patients suffered from chemotherapy resistance. The analysis of IHC staining implied that the chemotherapy resistance CRC patients had a higher expression of ATP7A protein than the chemotherapy response patients (**Figures 1A, B**, P<0.001). Our analysis also implied that the metastatic CRC patients (TNM Stage IV) had a higher expression of ATP7A in tumor tissue than locally advanced CRC patients (TNM Stage III, P<0.01). However, there was no statistical significance in survival (OS and DFS) between the higher and lower expression of ATP7A CRC patients (**Supplementary Figure 1**). The correlation between ATP7A expression and clinicopathologic features of CRC patients could be found in **Supplemental Table 1**.

Based on the GEPIA database, the bioinformatic analysis of ATP7A implied that there was no statistical significance between ATP7A high-expressed and low-expressed CRC patients in OS (**Supplemental Figure 2A**). However, the higher expression of ATP7A CRC may have a worse prognosis in DFS (**Supplemental Figure 2B**, HR=1.5, P=0.048). There was no ATP7A expression difference between the adjacent normal tissue and tumor tissue (**Supplemental Figure 2C**), and the bioinformatic analysis displayed that the ATP7A expression was not related to the TNM stage (**Supplemental Figure 2D**).

### ATP7A Inhibition Reverse OXA Resistance *In Vitro*

**Figure 2:**
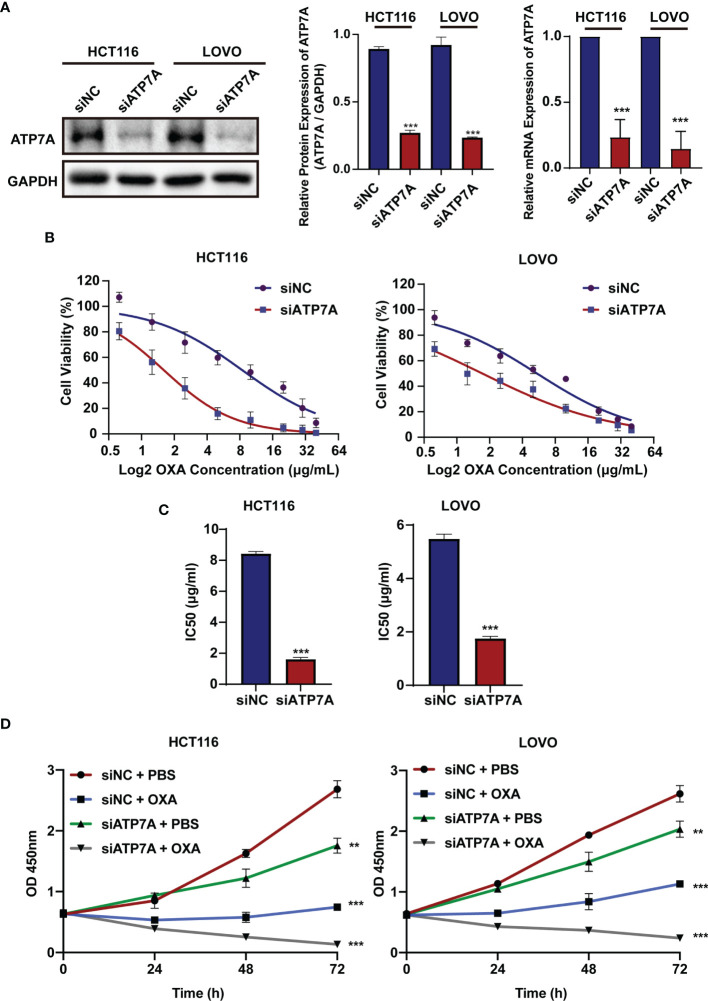
ATP7A inhibition reverse OXA resistance *in vitro*. **(A)** ATP7A protein and ATP7A mRNA expression were inhibited by the siATP7A in HCT116 and LOVO cell lines. **(B)** OXA sensitivity in HCT116 and LOVO cell lines treated with siATP7A was determined using the CCK-8 test after treatment with various concentrations of OXA for 48 h. **(C)** The IC50 of OXA for siNC and siRNA transfected HCT116 or LOVO cells. **(D)** Together with OXA, decreased expression of ATP7A significantly suppressed cancer cell lines proliferation. **P < 0.01 and ***P < 0.001 when compared to the control group.

To investigate the biological role of ATP7A in CRC, we knocked down ATP7A using siRNA. HCT116 and LOVO cell lines were transfected with siATP7A. The western blot and qPCR results revealed that in siATP7A-transfected HCT116 and LOVO cells, ATP7A was downregulated obviously (**Figure 2A**, P<0.001). To further research the effects of ATP7A in CRC cell chemosensitivity, ATP7A-knockdown CRC cells were treated with various concentrations of OXA for 48h, and the cytotoxic effects of OXA were measured using the CCK-8 assay (**Figure 2B**). When ATP7A was knocked down, the IC50 value of OXA in siATP7A-transfected HCT116 (IC50 = 1.604 μg/ml, 95% CI = 1.419 to 1.807 μg/ml) was substantially lower than that in siNC-transfected HCT116 (IC50 = 8.407 μg/ml, 95% CI = 6.883 to 10.24 μg/ml). Similarly, the IC50 value of OXA for siATP7A-transfected LOVO (IC50 = 1.713μg/ml, 95% CI = 1.389 to 2.071 μg/ml) was significantly lower than that of siNC-transfected LOVO (IC50 = 5.439μg/ml, 95% CI =4.653 to 6.340μg/ml, **Figure 2C**, P<0.001). Furthermore, the CCK-8 assay proved that the cell viability of ATP7A downregulation cells was reduced compared to the negative control (**Figure 2D**, P<0.001). The results indicated that ATP7A downregulation noticeably increased and resensitized the chemosensitivity of CRC cells to OXA.

### Knockdown of ATP7A Expression May Inhibit Cancer Tumorigenesis *In Vitro*

**Figure 3:**
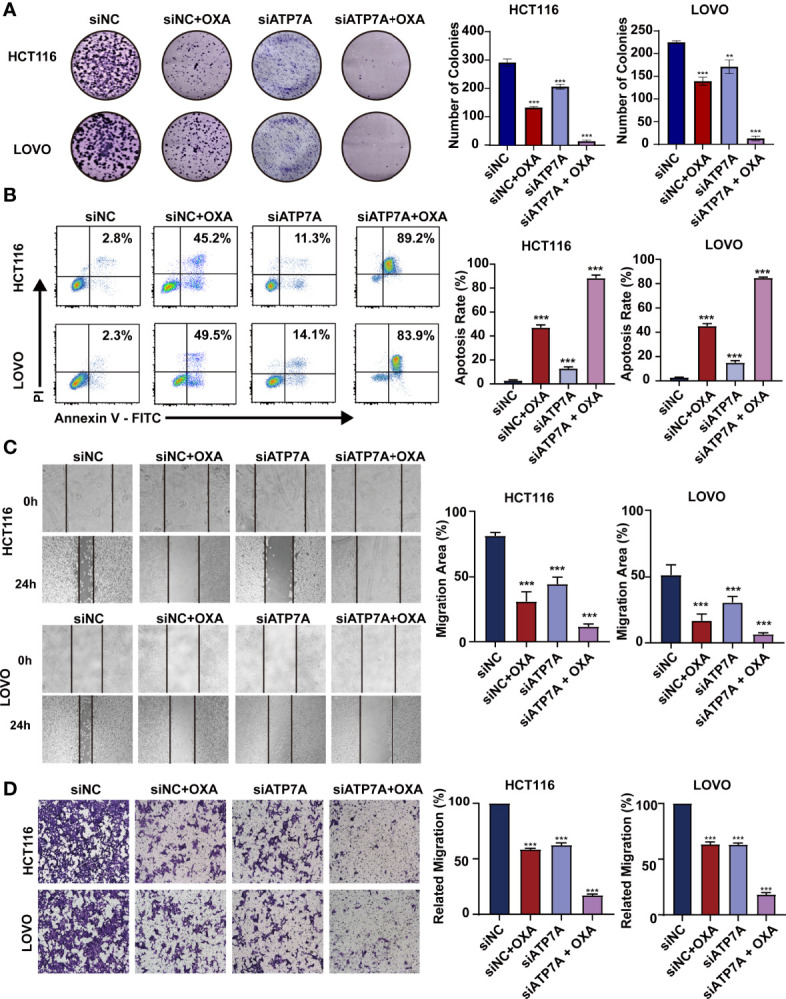
Knockdown of ATP7A expression may inhibit cancer tumorigenesis *in vitro*. **(A)** Colony formation assays in HCT116 and LOVO cells. **(B)** A flow cytometer was performed to assess the apoptosis rate of HCT116 and LOVO cells. **(C)** Wound healing assay of HCT116 and LOVO cells. **(D)** Transwell assay was performed to evaluate the invasion of HCT116 and LOVO cells. *P < 0.05; **P < 0.01 and ***P < 0.001 when compared to the control group.

The results of colony-forming assay showed that ATP7A knockdown significantly decreased the colony formation ability with OXA treatment (**Figure 3A**, P<0.001). As for the apoptosis detection by FACS, the results suggested that the combination of ATP7A knockdown and OXA treatment largely inhibited the cell growth and improved the ratio of late apoptosis (**Figure 3B** P<0.001). To assess the effect of ATP7A on CRC cell motility, we performed wound-healing assays and transwell assays. Both HCT116 and LOVO treated with siATP7A knockdown plus OXA show the decreased ability of migration compared with the control (**Figures 3C, D**, P<0.001).

### Construction of DAN Nanoparticle

**Figure 4:**
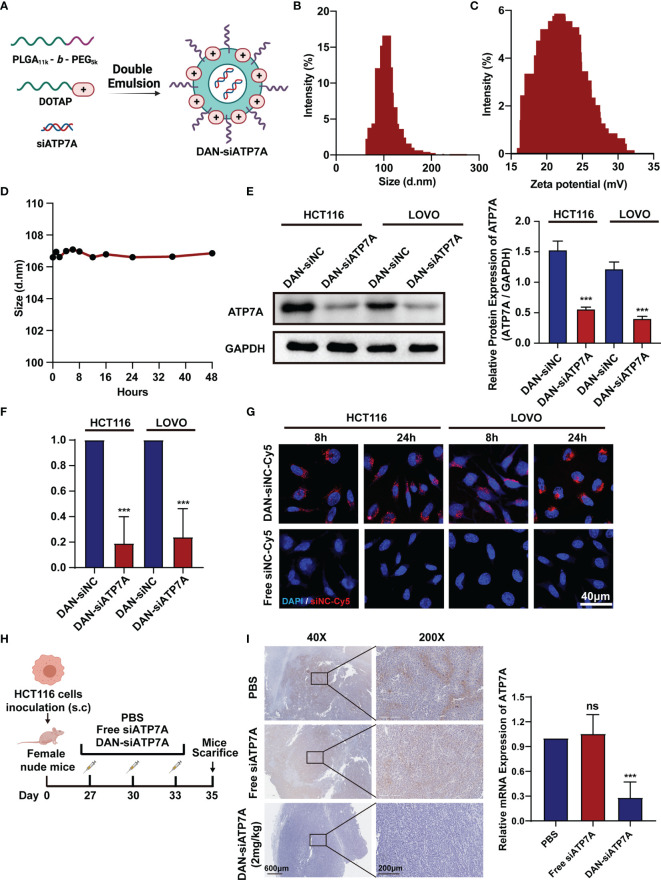
Construction of DAN nanoparticle. **(A)** The schematic illustration of the nanoparticle DAN-siATP7A construction. The size **(B)** and zeta potential **(C)** distribution of DAN-siATP7A. **(D)** The size measurement of DANsiATP7A in the RPMI-1640 supplemented with 10% FBS. The protein expression **(E)** and mRNA expression **(F)** of ATP7A after being transfected with DAN-siATP7A in HCT116 and LOVO cell lines. **(G)** The in Virto cellular uptake of Cy5-labeled DAN-siNC and free Cy5-labeled siNC *in vitro*. The confocal image was captured 8 hours and 24hours after the transfection of HCT116 and LOVO cell lines. **(H)** The scheme of medication on tumor-bearing mice. **(I)** The IHC analysis of ATP7A and the PCR analysis of ATP7A mRNA in tumor tissue implied effective knockdown of ATP7A *via* DAN-siATP7A nanoparticles. ***P < 0.001 and ns, not significance, when compared to the control group.

To deliver siATP7A effectively *in vitro* and *in vivo*, the copolymer PEG5K-b-PLGA11K and a cationic lipid (DOTAP) were chosen to encapsulate siATP7A *via* a double-emulsion method. The components used for DAN-siATP7A preparation are shown in **Figure 4A**. In addition, the diameter of DAN-siATP7A was 101.4 ± 13.84 nm (**Figure 4B**) and the zeta potential was 21.77 ± 3.26mV (**Figure 4C**). To evaluate the stability of DAN-siATP7A, we incubated it in PRIM-1640 with 10% FBS for 48 h and the size of DAN-siATP7A changed very slightly (**Figure 4D**), which implied that DAN-siATP7A was relatively stable in blood circulation. Meanwhile, we further investigate the DAN-siATP7A in the following *in vitro* and *in vivo* studies. After being transferred by DAN-siATP7A, the ATP7A protein expression and mRNA expression in CRC cells showed the same significant inhibition (**Figures 4E, F**, P<0.001). Further, we investigate the cellular uptake of DAN-siRNA. As shown in **Figure 4G**, confocal images indicated that Cy-5 labeled siRNA can be delivered into the CRC cells successfully by DAN over time. *In vivo* study, we evaluated the transfection efficiency in the colorectal cancer model of nude mice (**Figure 4H**). Then, the result of IHC showed that the DAN-siATP7A group displayed a significant reduction of ATP7A protein expression compared to the free siATP7A group (**Figure 4I**, P<0.001), indicating that DAN-siATP7A could be delivered into the CRC tissue and played a significant role in knocking down.

### siRNA-Loaded Nanoparticle Reverses Chemoresistance *In Vivo*

**Figure 5:**
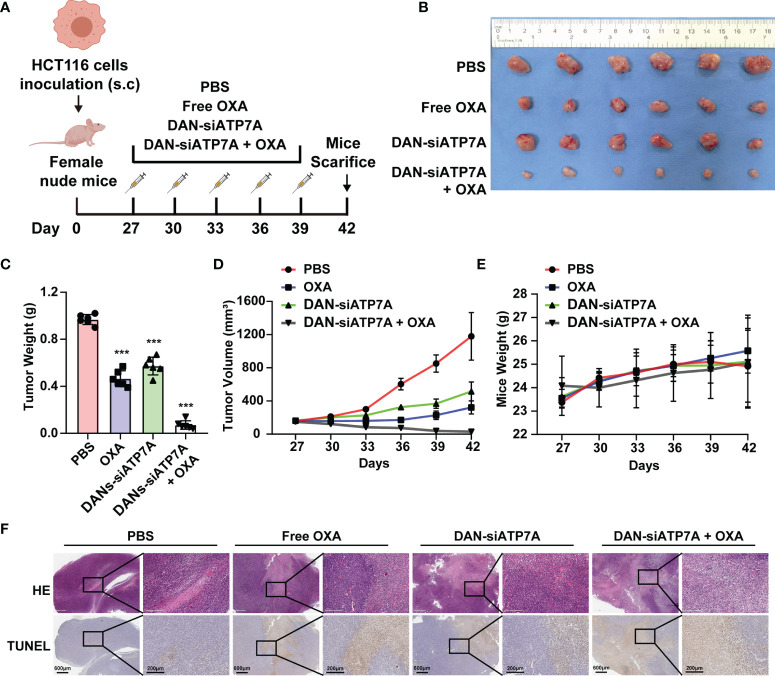
siRNA-loaded nanoparticle reverses chemoresistance in HCT116-bearing mice. **(A)** The scheme of medication on tumor-bearing mice. **(B)** The image of the tumor was dissected from the nude mice at the end of the *in vivo* study. Tumor weight **(C)**, tumor volume **(D)** and mice weight **(E)** after the treatment. **(F)** The HE staining and TUNEL analysis of the tumor tissue of the nude mice. ***P < 0.001 when compared to the control group.

A xenografted HCT116 model of human CRC in nude mice was established to investigate the therapeutic effect of different groups *in vivo* (**Figure 5A**). **Figures 5B, C** showed tumor images and weight curves. The tumor size treated in the combination group (OXA+DAN-siATP7A) was smaller compared with others: free OXA, free DAN-siATP7A, and control group. We also recorded the tumor volume changes at different time points to evaluate the therapeutic efficacy. As **Figure 5D** shown, the tumor volumes of mice after treatment in combination therapy were also much smaller (P < 0.001), indicating the increased combinational therapeutic efficacy and the reversal effect of DAN-siATP7A on OXA resistance *in vivo*. Furthermore, the quantitative analysis of TUNEL staining was applied to analyze the tumor apoptosis *in vivo* (**Figure 5F** and **Supplemental Figure 3**). The highest apoptosis rate was observed in the combination of DAN-siATP7A and OXA treatment groups. Similarly, H&E staining (**Figure 5F**) shows the same trend, indicating the reversal role of DAN-siATP7A on OXA resistance *in vivo* during the antitumor process.

### *In Vivo* Toxicity Analysis

**Figure 6:**
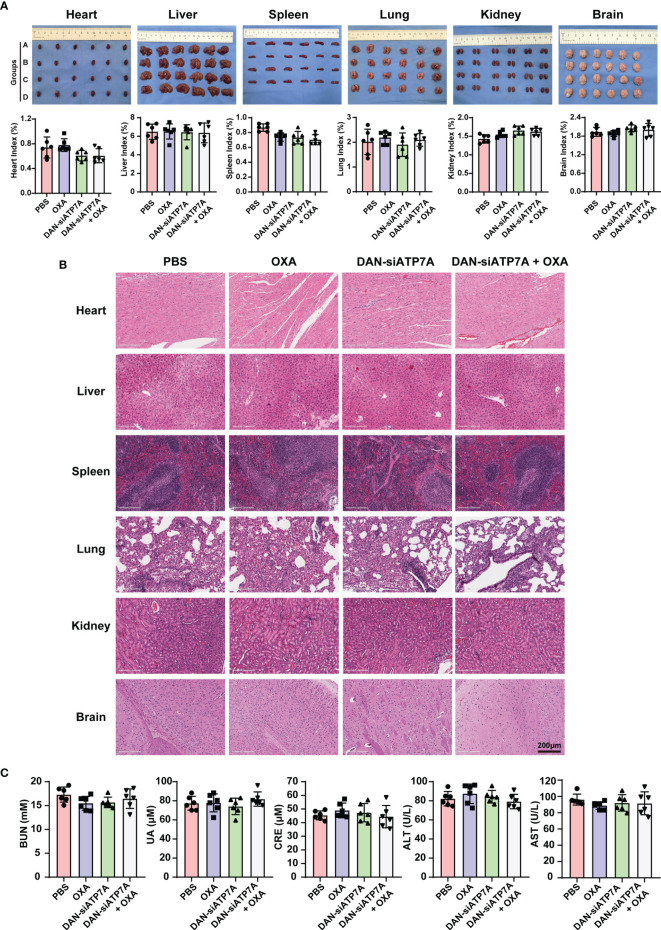
*In vivo* toxicity analysis. **(A)** The image of organs including heart, liver, spleen, lung, kidney, and brain from the nude mice at the end of the *in vivo* study and the organ index (organ index = the weight of the organ/the weight of the mice) of the nude mice. Data were expressed as means ± SD. Group A stands for PBS; Group B stands for OXA; Group C stands for DAN-siATP7A and Group D stands for DAN-ATP7A + OXA. **(B)** The HE staining of organs. **(C) **The blood test of ALT, AST, BUN, Cr, UC of the nude mice, and no toxicity were found in our *in vivo* experiment.

We also evaluated the systematic toxicity of OXA and DAN-siATP7A by monitoring the body weights of nude mice for 42 days. The body weight of the control, free OXA, and free DAN-siATP7A groups displayed increasing over time due to the increased tumor growth of the three groups. However, the body weights of the OXA and DAN-siATP7A groups did not seem to significantly change (**Figure 5E**), suggesting that the combination treatment do not have obvious toxicity to the mice. In addition, the main organs of mice were dissected, H&E stained, and observed by optical microscopy (**Figures 6A, B**). The organ index was calculated by the following formula: Organ Index(%) = [(the weight of the organ)/(the weight of the mice)] *100. As displayed in **Figure 6A**, there was no difference was found in each group of organ index. We also evaluate the potential hepatic and renal impairment from the blood sample of the nude mice, including the index of ALT, AST, GGT, BUN, Cr, and UC (**Figure 6C**). Our results indicated that no obvious adverse effects to the major organs of mice were observed after the mice were treated with the combination treatment of OXA and DAN-siATP7A.

## Discussion

Oxaliplatin-based chemotherapy is currently the first-line clinical treatment for advanced CRC patients. However, chemotherapy failure or resistance was the major cause of recurrence and poor prognosis in CRC patients. It is of great importance to identify the therapy targets and develop novel therapeutic strategies to overcome chemotherapy resistance to improve the survival of the patients.

Previous reports demonstrated that the mechanisms of OXA resistance include uptake and metabolic abnormalities, changes in the tumor microenvironment. Herein, enhanced drug efflux and decreased drug concentration both contributed to chemotherapy resistance, in which ATP-binding cassette (ABC) transporters were the most prominent (32–34). Previous researches found out that the up-regulated ATP7A was correlated to the platinum drug resistance in colon cancer, esophageal squamous cell cancer, gastric carcinoma, and ovarian cancer (14). In the research of esophageal squamous cell cancer, researchers found out that the ATP7A knockdown by siRNA partially reversed resistance, and may provide novel strategies to overcome platinum drug resistance (35).

Hence, in our study, we identified ATP7A as a potentially key gene of OXA resistance in CRC. In our cohorts, the patients with higher expression ATP7A tended to have platinum drug resistance. The lower expression of ATP7A by siRNA knockdown resulted in enhancement of OXA sensitivity and increased OXA-induced apoptosis. We also demonstrated a novel strategy to increase CRC chemosensitivity by delivering siRNAs into tumor cells by developing a novel nano-particles, DAN. The nanoparticles exhibit an ideal anti-tumor effect combined with the OXA *in vivo*. DAN nanoparticles were based on the FDA-approved material, PEG-PLGA (24). As a result, it displayed less *in vivo* toxicity in the mouse xenograft. In brief, we identified a key gene in CRC chemoresistance *via* clinical samples and *in vitro* experiments, and develop novel and effective nanoparticles to overcome the platinum drug resistance in colon cancer.

In the survival analysis, there was not enough evidence revealing that the lower-expression of ATP7A CRC patients benefit from a better clinical prognosis in our cohort (**Supplementary Figure 1**). To find out its cause, the results may be concluded from the limited samples from our cohorts (41 patients) and advanced CRC patients enrolled in our study may have poor prognosis than the early-stage CRC patients. Despite no prognosis difference in OS analysis, the bioinformatic analysis revealed that the lower ATP7A expressed CRC patients may have a better clinical prognosis in DFS (**Supplementary Figure 2B**, P<0.05). Due to the conflicted conclusion of survival analysis, we implied that the ATP7A may not act as a prognostic indicator for CRC patients.

We provided the pieces of clinical evidence that the ATP7A was upregulated in OXA-resistant CRC patients compared to those patients who respond to chemotherapy (**Figure 1**). The previous researches found out the higher expression of ATP7A was correlated with the chemotherapy resistance. Samimi G et al. demonstrated that after being transfected with an ATP7A-expression construct, the human ovarian carcinoma cells also conferred resistance to DDP, CBDCA, and OXA (36). As a result, we hypothesis that higher ATP7A expression may be correlated to the OXA-based chemoresistance in CRC patients. The lower ATP7A expression CRC patients may benefit from the chemotherapy. However, due to the limited sample size of our study, it could not be concluded that the ATP7A may be a key regulator of CRC chemoresistance, and all the results of clinical analysis has no clinical implications currently. A multi-center with larger cohorts study should be conducted further to support the conclusion.

In our study, the present results indicated that knockdown of ATP7A by siRNA enhanced OXA sensitivity and reversed chemoresistance both *in vitro* and *in vivo*. *In vitro*, increased chemosensitivity and apoptosis, and decreased cell proliferation were evident in CRC cells following siRNA-mediated knockdown of ATP7A, suggesting that ATP7A is one of the important biomarkers for OXA resistance in CRC. Further, we found that the combination of OXA and siATP7A could inhibit the migration of CRC cells (**Figures 3C, D**).

Moreover, the analysis of IHC staining implied that the chemotherapy resistance CRC patients had a higher expression of ATP7A protein than the chemotherapy response patients and the metastatic CRC patients (TNM Stage IV) had a higher expression of ATP7A in tumor tissue than locally advanced CRC patients (**Figure 1B**). Our findings provide evidence that decreased drug efflux by knockdown ATP7A in CRC cells could lead up to higher tissue distribution and intracellular drug accumulation to form destructive DNA-platinum adducts which ultimately destroy cancer cells. These results all suggested that ATP7A played a vital role in the modulation of Pt-resistance.

Since when genetic interference was discovered in 1998, siRNA has been recognized as a powerful genetic interference tool to specifically silence the target genes to tackle cancer angiogenesis, invasion, and metastasis (23). Oxaliplatin therapy might have unrealized clinical benefits due to a lack of an appropriate carrier delivering siATP7A. Unfortunately, it is a great challenge for delivery of siRNA to tumors *in vivo* in consideration of the barrier of the blood circulation and poor cellular uptake (20, 21). In the present study, our strategy may provide a solution to this challenge. We explore a novel nanocarrier-based delivery of ATP7A as a therapeutic strategy for nanoparticles-mediated chemotherapy sensitization, which was based on the PEG-PLGA and DOTAP (24). Specifically, we encapsulated siRNA targeting ATP7A using DANs and confirmed that DAN-siATP7A could be effectively enriched in tumor tissue and downregulate the target gene (**Figure 4**). The therapeutic efficacy of different forms *in vivo* was verified by the enhanced cell apoptosis rate and the enhanced tumor inhibition efficacy (**Figure 5**). In addition, the combination therapy is relatively safe as confirmed by the detection of various organs and blood indexes (**Figure 6**). Let it be added here that the composition of DANs, PEG-PLGA, is a pharmaceutically approved drug for clinical by Food and Drug Administration (FDA). Yet the biosecurity and anti-tumor effect of DAN-siATP7A in OXA-resistance patients is by far less well understood. Most of these data are derived from *in vitro* drug-exposed tumor cells and in nude mice, which have not yet entered the clinical stage and need further verifications.

In conclusion, our findings demonstrate that ATP7A may act as a therapeutic target for CRC patients. The delivery of siATP7A *via* nanoparticles increases the sensitivity of CRC cells to oxaliplatin, thereby providing a new treatment strategy for CRC. However, due to the limitations in our study, such as limited cohorts from single-center retrospective study, the conclusions of clinical analysis may only implied the higher expression of ATP7A may correlated with chemo-resistance in CRC patients. All the results from clinical analysis currently have no clinical implications and needed to be further validated.

## Data Availability Statement

The original contributions presented in the study are included in the article/**Supplementary Material**. Further inquiries can be directed to the corresponding authors.

## Ethics Statement

The studies involving human participants were reviewed and approved by Guangdong Provincial People’s Hospital. The patients/participants provided their written informed consent to participate in this study. The animal study was reviewed and approved by Guangdong Provincial People’s Hospital.

## Author Contributions

The study concept and design were carried out by all the authors who participate in the study. The experiments were performed by YZ, QZ, MW, and CH, YZ, QZ, and MW wrote the draft of the manuscript. All the authors conducted the critical revision of the manuscript and finally approved the version.

## Funding

This work was supported by grants from the Science and Technology Planning Project of Guangdong Provincial People’s Hospital (Guangdong Academy of Medical Sciences, No. DFJH201913), CSCO-Roche Cancer Research Foundation (No. Y-2019Roche-190), CSCO-Haosen Research Foundation (No. Y-HS2019/2-050), the Science and Technology Planning Project of Ganzhou (No. 202101074816).

## Conflict of Interest

The authors declare that the research was conducted in the absence of any commercial or financial relationships that could be construed as a potential conflict of interest.

## Publisher’s Note

All claims expressed in this article are solely those of the authors and do not necessarily represent those of their affiliated organizations, or those of the publisher, the editors and the reviewers. Any product that may be evaluated in this article, or claim that may be made by its manufacturer, is not guaranteed or endorsed by the publisher.
