# Corrigendum: Effective Delivery of siRNA-Loaded Nanoparticles for Overcoming Oxaliplatin Resistance in Colorectal Cancer

**DOI:** 10.3389/fonc.2022.916983

**Published:** 2022-06-27

**Authors:** Yue Zhou, Qing Zhang, Minjia Wang, Chengzhi Huang, Xueqing Yao

**Affiliations:** ^1^ The Second School of Clinical Medicine, Southern Medical University, Guangzhou, China; ^2^ Department of Gastrointestinal Surgery, Ganzhou Municipal Hospital, Ganzhou, China; ^3^ Department of Gastrointestinal Surgery, Department of General Surgery, Guangdong Provincial People’s Hospital, Guangdong Academy of Medical Sciences, Guangzhou, China; ^4^ Department of Gastrointestinal and Anorectal Surgery, The First People’s Hospital of Zhaoqing, Zhaoqing, China; ^5^ School of Medicine, South China University of Technology, Guangzhou, China

**Keywords:** oxaliplatin, chemoresistance, siRNA delivery, colorectal cancer, ATP7A

In the original article, there was a mistake in **Figure 3** as published. While preparing the figures, the labels of the bar charts in **Figure 3** were misspelled. The corrected **Figure 3** appears below.

**Figure 3:**
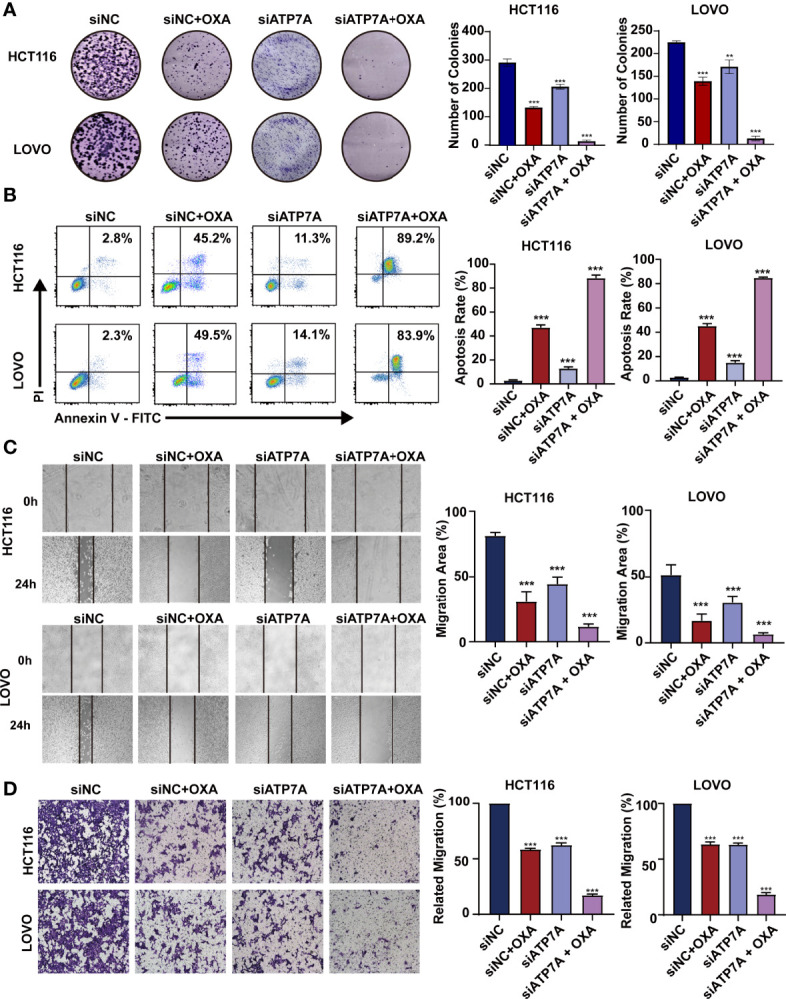
Knockdown of ATP7A expression may inhibit cancer tumorigenesis *in vitro* **(A)** Colony formation assays in HCT116 and LOVO cells. **(B)** A flow cytometer was performed to assess the apoptosis rate of HCT116 and LOVO cells. **(C)** Wound healing assay of HCT116 and LOVO cells. **(D)** Transwell assay was performed to evaluate the invasion of HCT116 and LOVO cells. **P < 0.01 and ***P < 0.001 when compared to the control group.

In the published article, there was also an error in affiliation 3. Instead of “Department of Gastrointestinal Surgery, Department of General Surgery, Guangdong Provincial People’s Hospital, Guangdong Academy of Medical Sciences, School of Medicine, South China University of Technology, Guangzhou, China”, it should be “Department of Gastrointestinal Surgery, Department of General Surgery, Guangdong Provincial People’s Hospital, Guangdong Academy of Medical Sciences, Guangzhou, China”.

There was a further error as an affiliation was omitted for authors Minjia Wang, Chengzhi Huang and Xueqing Yao. These authors should also have affiliation 5 – “School of Medicine, South China University of Technology, Guangzhou, China”.

The authors apologize for these errors and state that this does not change the scientific conclusions of the article in any way. The original article has been updated.

## Publisher’s Note

All claims expressed in this article are solely those of the authors and do not necessarily represent those of their affiliated organizations, or those of the publisher, the editors and the reviewers. Any product that may be evaluated in this article, or claim that may be made by its manufacturer, is not guaranteed or endorsed by the publisher.

