# Supplementary material for: Effective Delivery of siRNA-Loaded Nanoparticles for Overcoming Oxaliplatin Resistance in Colorectal Cancer
Source: Front Oncol. 2022 Feb 21;12:827891. doi: 10.3389/fonc.2022.827891 (PMC8898837; doi:10.3389/fonc.2022.827891)
Supplement: Supplementary file 1 [file DataSheet_1.pdf]

Supplementary Figures and Tables for

Effective delivery of siRNA-loaded nanoparticles for overcoming oxaliplatin resistance in colorectal cancer

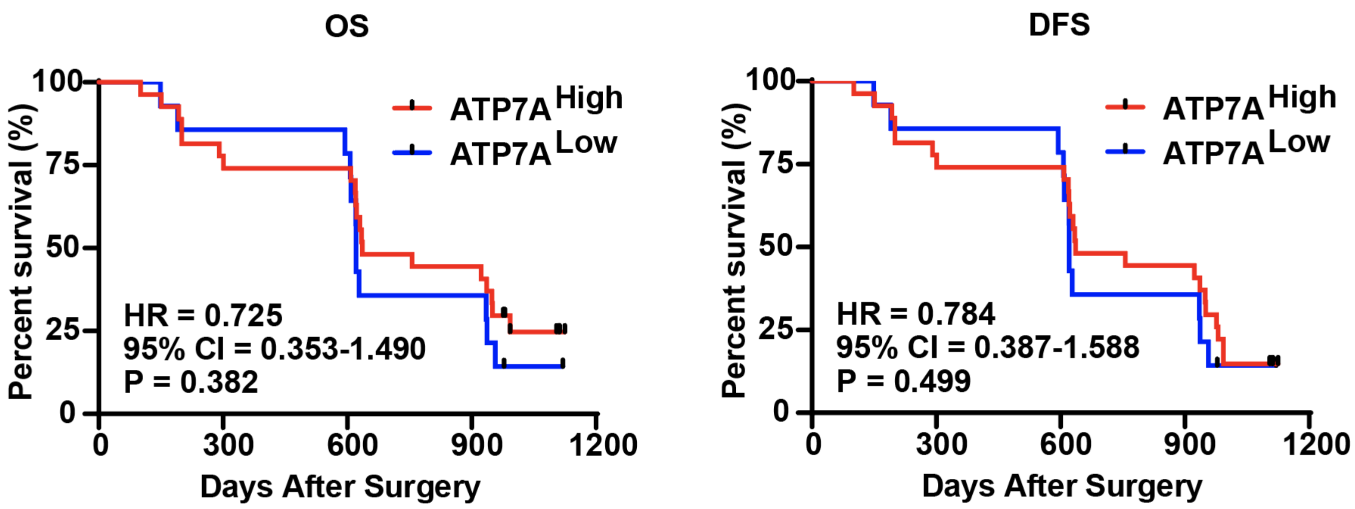

Supplementary Figure 1 | The prognosis analysis of ATP7A in CRC.

The Kaplan-Meier analysis of the OS and the DFS of CRC patients with low or high expression of ATP7A.

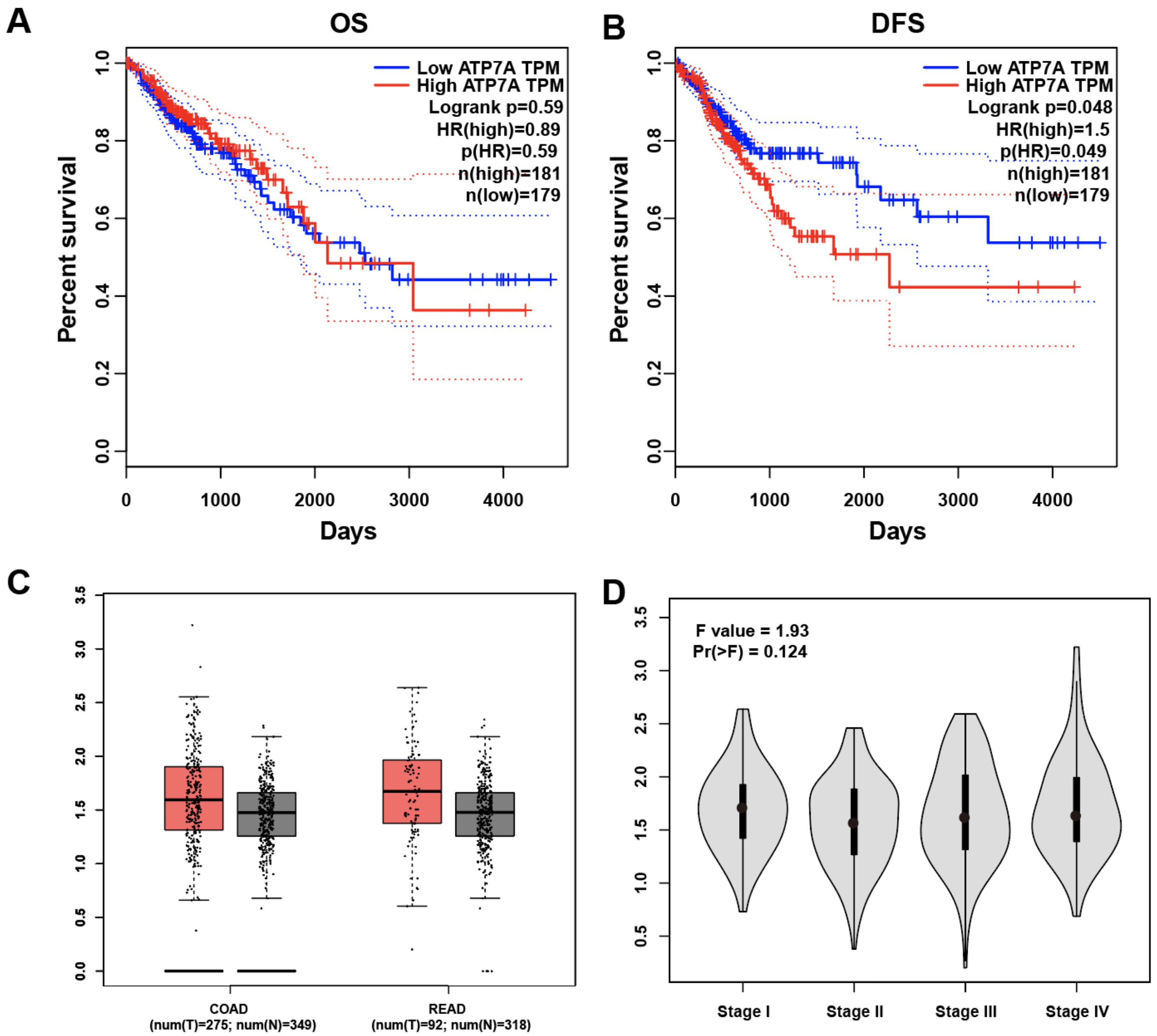

Supplementary Figure 2 | The bioinformatics analysis of ATP7A and CRC via GEPIA database.

(A) The expression of ATP7A may not correlate with the OS. (B) The higher-expressed ATP7A implied poor DFS in CRC patients. (C) The ATP7A expression displayed no difference between tumor tissue and normal tissue. (D) The ATP7A expression was not correlated with the TNM stage of CRC patients in the TCGA cohort

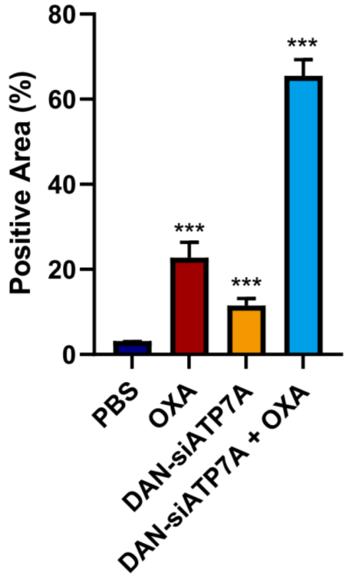

Supplementary Figure 3 | The quantitative analysis of TUNEL staining.

*, $P<0.05$; **, $P<0.01$ and ***, $P<0.001$ when compared to the control group.

Group	n	%	ATP7A Expression					
			Low expression	%	High Expression	%	Chi-squre	P
Total	41							
**Gender**								
Female	26	63.41	9	64.29	17	62.96	0.007	0.934
Male	15	36.59	5	35.71	10	37.04		
**Age**								
⩽60	14	34.15	5	35.71	9	33.33	0.023	0.879
>60	27	65.85	9	64.29	18	66.67		
**Tumor Location**								
Colon	24	58.54	10	71.43	14	51.85	1.456	0.228
Rectum	17	41.46	4	28.57	13	48.15		
**Tumor Stage (TNM Stage)**								
III (Local Advanced)	32	78.05	14	100.00	18	66.67	5.979	0.014
IV (Distant Metastasis)	9	21.95	0	0.00	9	33.33		
**Tumor Differentation**								
Median or Well	16	39.02	4	28.57	12	44.44	0.976	0.323
Poor	25	60.98	10	71.43	15	55.56		
**Chemotherapy Resistance**								
Response	20	48.78	13	92.86	7	25.93	16.53	<0.01
Resistance	21	51.22	1	7.14	20	74.07		

Supplementary Table 1 | Correlation between MGP expression and clinicopathologic features of CRC patients.
